# Second-dose ChAdOx1 and BNT162b2 COVID-19 vaccines and thrombocytopenic, thromboembolic and hemorrhagic events in Scotland

**DOI:** 10.1038/s41467-022-32264-6

**Published:** 2022-08-15

**Authors:** Colin R. Simpson, Steven Kerr, Srinivasa Vittal Katikireddi, Colin McCowan, Lewis D. Ritchie, Jiafeng Pan, Sarah J. Stock, Igor Rudan, Ruby S. M. Tsang, Simon de Lusignan, F. D. Richard Hobbs, Ashley Akbari, Ronan A. Lyons, Chris Robertson, Aziz Sheikh

**Affiliations:** 1 School of Health, Wellington Faculty of Health, Victoria University of Wellington, Wellington, New Zealand; 2 Usher Institute, The University of Edinburgh, Edinburgh, UK; 3 Public Health Scotland, Edinburgh, UK; 4 MRC/CSO Social & Public Health Sciences Unit, University of Glasgow, Glasgow, UK; 5 School of Medicine, University of St Andrews, St Andrews, UK; 6 Centre of Academic Primary Care, University of Aberdeen, Aberdeen, UK; 7 Department of Mathematics and Statistics, University of Strathclyde, Strathclyde, UK; 8 Nuffield Department of Primary Care Health Sciences, University of Oxford, Oxford, UK; 9 Population Data Science, Swansea University, Swansea, UK; 10 Health Data Research UK, BREATHE Hub, London, UK

**Keywords:** Viral infection, Epidemiology, SARS-CoV-2, Vaccines

## Abstract

We investigated thrombocytopenic, thromboembolic and hemorrhagic events following a second dose of ChAdOx1 and BNT162b2 using a self-controlled case series analysis. We used a national prospective cohort with 2.0 million(m) adults vaccinated with two doses of ChAdOx or 1.6 m with BNT162b2. The incidence rate ratio (IRR) for idiopathic thrombocytopenic purpura (ITP) 14–20 days post-ChAdOx1 second dose was 2.14, 95% confidence interval (CI) 0.90–5.08. The incidence of ITP post-second dose ChAdOx1 was 0.59 (0.37–0.89) per 100,000 doses. No evidence of an increased risk of CVST was found for the 0–27 day risk period (IRR 0.83, 95% CI 0.16 to 4.26). However, few (≤5) events arose within this risk period. It is perhaps noteworthy that these events all clustered in the 7–13 day period (IRR 4.06, 95% CI 0.94 to 17.51). No other associations were found for second dose ChAdOx1, or any association for second dose BNT162b2 vaccination. Second dose ChAdOx1 vaccination was associated with increased borderline risks of ITP and CVST events. However, these events were rare thus providing reassurance about the safety of these vaccines. Further analyses including more cases are required to determine more precisely the risk profile for ITP and CVST after a second dose of ChAdOx1 vaccine.

## Abstract (web summary)

Here, Simpson et al. analyze data from 3.6 million COVID-19 vaccine second doses (ChAdOx1 and BNT162b2) in Scotland for risk of thrombocytopenic, thromboembolic and hemorrhagic events. Borderline increased risks of immune thrombocytopenic purpura and cerebral venous sinus thrombosis were found for the ChAdOx1 vaccine. These events were rare and usually short-lived.

## Introduction

The three Coronavirus Disease 2019 (COVID-19) vaccines currently being administered in the United Kingdom (UK), namely ChAdOx1 nCoV-19 (Oxford-AstraZeneca; henceforth ChAdOx1), BNT162b2 mRNA (Pfizer-BioNTech; henceforth BNT162b2) and mRNA-1273 (Moderna), have been shown to reduce COVID-19 infection, hospitalization and death^1–4^. The mRNA-1273 vaccine was first given in Scotland on 7 April 2021 and remains a less commonly used vaccine (169,842 second doses as of 13 January 2022)^5^.

There have been reports of post-vaccination exacerbation of chronic idiopathic or immune thrombocytopenic purpura (ITP) amongst those receiving mRNA vaccines (including BNT162b2 and ChAdOx1)^6–9^. ITP associated with first dose ChAdOx1 vaccine has previously been reported using the Early Pandemic Evaluation and Enhanced Surveillance of COVID-19 (EAVE II) surveillance platform^10–12^ and based on our analysis and other evidence, ITP was added to the product information as a side-effect of ChAdOx1 on 6 October 2021^13^. There have also been reports of adverse events following first dose ChAdOx1 vaccination including vaccine induced immune thrombotic thrombocytopenia (VITT) and thrombosis with thrombocytopenia syndrome (TTS) resulting in venous or arterial thrombosis, including cerebral venous sinus thrombosis (CVST), and thrombocytopenia^7–9^.

There is the need for evidence on the safety of the second dose COVID-19 vaccines and, in particular, any association with ITP, venous thromboembolic (including CVST), arterial thromboembolic and hemorrhagic events described in case reports^7^. To investigate this, we used a national prospective COVID-19 surveillance cohort in Scotland, which consisted of linked databases containing individual-level patient data relating to vaccination status, virological real-time reverse transcriptase polymerase chain reaction (RT-PCR) COVID-19, laboratory tests, and clinical and mortality records.

## Results

Between 8 December 2020 and 7 November 2021, 2.0 million people (39,682 females and 33,588 males under 30 years of age) were vaccinated with a second dose of ChAdOx1 and 1.6 million people (294,511 females and 276,476 males under 30 years of age) with a second dose of BNT162b2 (Extended Data Fig. 1).

### Thrombocytopenia

No increased risk of thrombocytopenia was found following second dose ChAdOx1 (0–27 days incidence rate ratio (IRR) = 0.84; 95% CI 0.62–1.13) (Table 1) or BNT162b2 vaccine (0–27 days IRR = 0.95; 95% CI 0.65–1.38) during any of the post-vaccination time periods (Table 2).

**Table 1:** Reported thrombocytopenic events for second dose ChAdOx1 vaccine

Time-period post vaccination	Number of events	Number of person-days	Incidence rate ratio (95% confidence intervals)
**Thrombocytopenia**			
Baseline^a^	443	152,498	1.00
Clearance^b^	25	8344	0.72 (0.47–1.09)
0–6 days	14	4897	0.82 (0.48–1.42)
7–13 days	19	4884	0.93 (0.56–1.56)
14–20 days	16	4876	0.64 (0.35–1.18)
21–27 days	17	4865	0.96 (0.57–1.60)
0–27 days	66	19,522	0.84 (0.62–1.13)
***Idiopathic thrombocytopenic purpura***			
Baseline^a^	85	33,948	1.00
Clearance^b^	8	1841	1.55 (0.72–3.36)
0–6 days	≤5	917	0.73 (0.18–3.05)
7–13 days	≤5	917	1.06 (0.33–3.45)
14–20 days	6	917	2.14 (0.90–5.08)
21–27 days	≤5	916	0.67 (0.16–2.80)
0–27 days	13	3667	1.15 (0.61–2.15)

^a^Includes all three baseline periods before dose 1 and before and after dose 2—see Fig. 1.

^b^Second dose clearance period NB ≤5 Denotes minimum allowable reported value.

**Table 2:** Reported thrombocytopenic events for second dose BNT162b2 vaccine

Time-period post vaccination	Number of events	Number of person-days	Incidence rate ratio (95% confidence intervals)
**Thrombocytopenia**			
Baseline^a^	256	88,315	1.00
Clearance^b^	12	4732	0.68 (0.38–1.23)
0–6 days	10	2394	1.09 (0.58–2.08)
7–13 days	8	2392	0.89 (0.44–1.82)
14–20 days	8	2386	0.88 (0.43–1.79)
21–27 days	8	2369	0.91 (0.45–1.86)
0–27 days	34	9541	0.95 (0.65–1.38)
***Idiopathic thrombocytopenic purpura***			
Baseline^a^	48	15,797	1.00
Clearance^b^	≤5	840	0.35 (0.05–2.60)
0–6 days	≤5	420	1.59 (0.38–6.71)
7–13 days	≤5	420	2.25 (0.68–7.44)
14–20 days	≤5	419	0.72 (0.10–5.26)
21–27 days	≤5	413	2.18 (0.66–7.16)
0–27 days	9	1672	1.68 (0.80–3.52)

^a^Includes all three baseline periods before dose 1 and before and after dose 2—see Fig. 1.

^b^Second dose clearance period. NB ≤5 Denotes minimum allowable reported value.

#### Idiopathic thrombocytopenic purpura

The incidence of ITP post-second dose ChAdOx1 was 0.59 per 100,000 doses (95% CI 0.37–0.89). The IRR 14–20 days post-vaccination for second dose ChAdOx1 was 2.14 (95% CI 0.90–5.08) (Table 1). The 0–27 days IRR was 1.15 (95% CI 0.61–2.15).

For patients who had ITP post-second dose ChAdOx1, five of thirteen patients (39%) were hospitalized with a median length of stay of one day (IQR 1–2 days). In comparison to the unvaccinated, these post-second dose patients were equally likely to have been hospitalized at the time of the event (39% vs. 25%, *p* = 0.493) and more likely to have at least one clinical risk condition (75% vs. 37%, *p* = 0.035). There were no deaths reported after post-second dose ITP. The sex and age distributions were similar for ITP after second dose ChAdOx1 vaccination compared to those who were unvaccinated: females 58% vs. 53%, *p* = 0.961) with a median age of 63 years (interquartile range (IQR) 53–76) vs. 54 years old (IQR 35–71).

All patients with a post-second dose vaccination ITP had platelet counts available, all 13 (100%) had counts below 150,000/µl and nine (69%) had counts below 100,000/µl. Sixty-five percent of post-second dose ChAdOx1 ITP cases had prior prescriptions that could induce drug dependent thrombocytopenia or ITP, compared with 51% of those who were unvaccinated at the time of their ITP event. Twenty four percent of ITP cases were prescribed ITP therapies by general practitioners in the community after vaccination with ChAdOx1.

No positive association was found between the BNT162b2 vaccine and ITP (Table 2) at any time-period (0–27 days: 1.68; 95% CI 0.80–3.52).

### Venous thromboembolic events

No increase in the IRR of venous thromboembolic events was found during any of the post-second dose vaccination time periods analyzed for ChAdOx1 with a 0–27 day IRR = 0.83 (95% CI 0.69–1.00) (Table 3) or BNT162b2 with a 0–27 day IRR = 0.80; 95% CI 0.69–0.94 (Table 4).

**Table 3:** Reported venous thromboembolic events for second dose ChAdOx1 vaccine

Time-period post vaccination	Number of events	Number of person-days	Incidence rate ratio (95% confidence intervals)
**Venous thromboembolic events**			
Baseline^a^	4476	1,461,642	1.00
Clearance^b^	162	80,498	0.57 (0.48–0.67)
0–6 days	93	40,618	0.66 (0.54–0.82)
7–13 days	123	40,535	0.89 (0.74–1.07)
14–20 days	121	40,382	0.89 (0.74–1.07)
21–27 days	130	40,259	0.97 (0.81–1.16)
0–27 days	467	161,794	0.83 (0.69–1.00)
***Cerebral venous sinus thrombosis***			
Baseline^a^	19	6986	1.00
Clearance^b^	≤5	378	3.80 (1.12–12.93)
0–6 days	0	189	0.00 (NA)
7–13 days	≤5	188	4.06 (0.94–17.51)
14–20 days	0	182	0.00 (NA)
21–27 days	0	177	0.00 (NA)
0–27 days	≤5	736	0.83 (0.16–4.26)
***Deep vein thrombosis***			
Baseline^a^	1808	599,137	1.00
Clearance^b^	70	32,932	0.56 (0.44–0.72)
0–6 days	46	16,629	0.76 (0.56–1.02)
7–13 days	52	16,605	0.87 (0.66–1.15)
14–20 days	62	16,549	1.05 (0.81–1.37)
21–27 days	49	16,489	0.84 (0.63–1.13)
0–27 days	209	66,272	0.88 (0.75–1.03)

*NA* Not Applicable as when there are 0 events, the CI cannot be calculated using conditional Poisson regression.

**Fig. 1:**
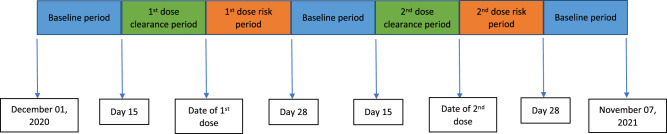
Schematic presentation of the self-controlled case series study design. The self-controlled case series study design—for each outcome of interest—risk periods were compared relative to baseline periods. Blue—Baseline period; Green—Clearance period; Orange—Risk period.

^a^Includes all three baseline periods before dose 1 and before and after dose 2—see Fig. 1.

^b^Second dose clearance period NB ≤5 Denotes minimum allowable reported value.

**Table 4:** Reported venous thromboembolic events for second dose BNT162b2 vaccine

Time-period post vaccination	Number of events	Number of person-days	Incidence rate ratio (95% confidence intervals)
**Venous thromboembolic events**			
Baseline^a^	1830	593,391	1.00
Clearance^b^	86	32,022	0.75 (0.60–0.93)
0–6 days	37	16,351	0.64 (0.46–0.89)
7–13 days	42	16,305	0.74 (0.54–1.00)
14–20 days	57	16,252	1.00 (0.77–1.31)
21–27 days	46	16,164	0.83 (0.62–1.12)
0–27 days	182	65,072	0.80 (0.69–0.94)
***Cerebral venous sinus thrombosis***			
Baseline^a^	≤5	1548	1.00
Clearance^b^	≤5	84	3.95 (0.37–42.26)
0–6 days	0	42	0.00 (NA)
7–13 days	0	42	0.00 (NA)
14–20 days	0	42	0.00 (NA)
21–27 days	0	42	0.00 (NA)
0–27 days	0	168	0.00 (NA)
***Deep vein thrombosis***			
Baseline^a^	736	244,086	1.00
Clearance^b^	43	13,178	0.90 (0.66–1.23)
0–6 days	17	6698	0.70 (0.43–1.13)
7–13 days	15	6685	0.62 (0.37–1.03)
14–20 days	20	6660	0.83 (0.53–1.30)
21–27 days	19	6615	0.81 (0.51–1.28)
0–27 days	71	26,658	0.74 (0.57–0.95)

*NA* Not Applicable as when there are 0 events, the CI cannot be calculated using conditional Poisson regression.

^a^Includes all three baseline periods before dose 1 and before and after dose 2—see Fig. 1.

^b^Second dose clearance period. NB ≤5 Denotes minimum allowable reported value.

#### Cerebral venous sinus thrombosis

When focusing on CVST after second dose ChAdOx1, the incidence of CVST was 0.05 per 100,000 doses (95% CI 0.01–0.19). The IRR 7–13 days post-vaccination was 4.06 (95% CI 0.94–17.51) (Table 3). For second dose ChAdOx1 vaccination associated CVST, all individuals were female, their mean age was 62 (IQR 58–65) and all had a hospital admission recorded at the time of the event with a median length of stay of 8 days (IQR 4–11 days). No individuals with a second dose vaccination CVST event died. Platelet count results (available for all individuals) were >150,000/µL.

No increased risk of CVST events was found for the BNT162b2 vaccine during any of the post-vaccination time periods (0–27 days IRR = 0.00) (Table 4).

#### Deep vein thrombosis and pulmonary embolism

An a priori subgroup analysis of deep vein thrombosis (DVT) and pulmonary embolism (PE) found no clear association with ChAdOx1 (Tables 3, 5) or BNT162b2 vaccination (Tables 4, 6) at any time post-second dose.

**Table 5:** Reported pulmonary embolism events for second dose ChAdOx1 vaccine

Time-period post vaccination	Number of events	Number of person-days	Incidence rate ratio (95% confidence intervals)
**Pulmonary embolism**			
Baseline^a^	2395	764,958	1.00
Clearance^b^	73	42,305	0.52 (0.41–0.66)
0–6 days	44	21,357	0.63 (0.47–0.86)
7–13 days	65	21,299	0.94 (0.73–1.21)
14–20 days	52	21,208	0.77 (0.58–1.01)
21–27 days	68	21,150	1.02 (0.79–1.30)
0–27 days	229	85,014	0.84 (0.72–0.97)

^a^Includes all three baseline periods before dose 1 and before and after dose 2—see Fig. 1.

^b^Second dose clearance period.

**Table 6:** Reported pulmonary embolism events for second dose BNT162b2 vaccine

Time-period post vaccination	Number of events	Number of person-days	Incidence rate ratio (95% confidence intervals)
**Pulmonary embolism**			
Baseline^a^	943	301,362	1.00
Clearance^b^	40	16,279	0.71 (0.51–0.98)
0–6 days	16	8362	0.56 (0.34–0.92)
7–13 days	23	8332	0.82 (0.54–1.25)
14–20 days	32	8305	1.16 (0.81–1.66)
21–27 days	25	8262	0.93 (0.62–1.39)
0–27 days	229	85,014	0.86 (0.69–1.08)

^a^Includes all three baseline periods before dose 1 and before and after dose 2—see Fig. 1.

^b^Second dose clearance period.

### Arterial thromboembolic events

The IRR for second dose ChAdOx1 and arterial thromboembolic events 0–27 days post-vaccination was 0.92 (95% CI 0.87–0.97) (Table 7). There was also no 0–27 days post-vaccination increased risk of arterial thromboembolic events associated with BNT162b2 vaccination (IRR = 0.86; 95% CI 0.79–0.93) or during any of the post-vaccination time periods analyzed for either vaccine (Tables 7, 8).

**Table 7:** Reported arterial thromboembolic and hemorrhagic events for second dose ChAdOx1 vaccine

Time-period post vaccination	Number of events	Number of person-days	Incidence rate ratio (95% confidence intervals)
**Arterial thromboembolic events**			
Baseline^a^	13,895	4,747,305	1.00
Clearance^b^	723	259,878	0.79 (0.73–0.86)
0–6 days	372	130,467	0.81 (0.73–0.90)
7–13 days	421	130,259	0.92 (0.83–1.02)
14–20 days	451	129,889	0.98 (0.89–1.08)
21–27 days	437	129,563	0.96 (0.87–1.05)
0–27 days	1681	520,178	0.92 (0.87–0.97)
**Hemorrhagic events**			
Baseline^a^	1320	435,077	1.00
Clearance^b^	64	23,973	0.71 (0.55–0.93)
0–6 days	30	12,076	0.66 (0.45–0.95)
7–13 days	40	12,034	0.88 (0.64–1.21)
14–20 days	35	11,985	0.78 (0.55–1.10)
21–27 days	45	11,923	1.02 (0.75–1.39)

^a^Includes all three baseline periods before dose 1 and before and after dose 2—see Fig. 1.

^b^Second dose clearance period NB ≤5 Denotes minimum allowable reported value.

**Table 8:** Reported arterial thromboembolic and hemorrhagic events for second dose BNT162b2 vaccine

Time-period post vaccination	Number of events	Number of person-days	Incidence rate ratio (95% confidence intervals)
**Arterial thromboembolic events**			
Baseline^a^	6225	2,095,989	1.00
Clearance^b^	319	118,274	0.78 (0.70–0.88)
0–6 days	173	59,234	0.78 (0.67–0.92)
7–13 days	183	59,087	0.96 (0.83–1.11)
14–20 days	168	58,877	0.83 (0.71–0.97)
21–27 days	188	58,616	0.85 (0.73–1.00)
0–27 days	710	229,727	0.86 (0.79–0.93)
**Hemorrhagic events**			
Baseline^a^	574	182,577	1.00
Clearance^b^	17	10029	0.44 (0.27–0.72)
0–6 days	13	5091	0.66 (0.38–1.15)
7–13 days	16	5088	0.81 (0.49–1.33)
14–20 days	16	5075	0.81 (0.49–1.34)
21–27 days	18	5043	0.92 (0.57–1.48)
0–27 days	63	20,297	0.80 (0.61–1.04)

^a^Includes all three baseline periods before dose 1 and before and after dose 2—see Fig. 1.

^b^Second dose clearance period.

### Hemorrhagic events

We found no clear evidence of associations between ChAdOx1 (0–27 days IRR = 0.83; 95% CI 0.69–1.00) and hemorrhagic events (Table 1). There was no 0–27 day post-vaccination increased risk of hemorrhagic events associated with BNT162b2 vaccination (IRR = 0.80; 95% CI 0.61–1.04) or during any of the post-vaccination time periods analyzed for either vaccine (Tables 7, 8).

## Discussion

This population-based analysis has found borderline increased risks of ITP and CVST following second dose ChAdOx1. There was no evidence of an association with any of the adverse events of interest after second dose BNT162b2. Several IRRs suggest a statistically significantly protective effect for ChAdOx1 and BNT262b2 vaccines. This includes VTE, PE, arterial thromboembolic, and hemorrhagic events for ChAdOx1; and VTE, DVT, PE, and arterial thromboembolic events for BNT262b2. This is most likely due to well-vaccinee bias, as people who are unwell defer vaccination. Alternatively, this might conceivably be a by-product of vaccine protection against COVID-19, if COVID-19 is a cause of these events^14^.

To our knowledge, this is the one of the first real-world contemporaneous studies identifying all individuals vaccinated with a second dose of COVID-19 vaccine within a national population assessing a range of hematological and vascular events. Previous studies have observed increased rates of thrombocytopenia, venous thromboembolic events, including CVST and intracerebral hemorrhage, after a first dose^14–16^. One French study found no significant increased risk in those over 75 years 14 days following first or second dose of BNT162b2 vaccine^17^. The incidence of ITP post 0–28 day second dose ChAdOx1 was lower than post first dose (0.59 vs. 1.13 per 100,000 doses)^12^. Post-second dose ChAdOx1 incident CVST events were similar (0.05 per 100,000 doses vs. 0.44 to 0.59 incident CVST cases per million people)^16^.

Our study has several strengths, including our ability to rapidly access and analyze data on vaccination status and medical and death records from linked national databases^10, 18^. This study is therefore less susceptible to recall or misclassification bias than studies of samples of the population. We believe our findings are likely to have generalizability across countries using these vaccines as part of national vaccination programs (with the proviso that further work is needed to determine if low adverse events rates in low- and middle-income countries is a result of underreporting or different susceptibility).

Study limitations include our reliance on diagnostic coding from the electronic health record (EHR) with possible under ascertainment of CVST^19^. Major diagnoses are likely to be recorded in a timely way, be accurate and have high completeness^20^. Information on the PPV and accuracy of timing for Read codes was not available. If low PPV occurs, it is likely to have biased results towards the null. Also, the EAVE II platform is a national public health surveillance platform that was established at the request of the Scottish Government to help inform the public health response to the pandemic. As the policy aim was for national coverage, it was not feasible to obtain individual patient consent. This therefore restricted our ability to interrogate and report on more detailed aspects of the clinical record. Due to delays in reporting of coded data, we did not use hospital coded data for our primary analysis^21^. Although there was a priori evidence that ITP might be clustered between 14 to 20 and 21 to 27 days (with incidence rate ratios of 7.81 and 14.07 respectively^12^) and unusual venous thrombosis events take place 5 to 16 day post ChAdOx1^7^, low numbers distributed across the time periods mean that this distribution could be a chance effect. Further investigation in studies with greater power is therefore warranted. SARS-CoV-2 infections were included in the analysis as time-varying confounders. It was also not possible to determine whether VITT/TTS was the underlying cause of CVST as we do not have access to anti-PF4 antibody data nor are we able to access platelet counts from the hospital data (all CVST were hospitalized for at least a week at the time of the event). The timing of reported ITP side-effects (on 9 June 2021)^12^ and age restrictions for ChAdOx1 (restricted to >30 years old on 7 April 2021 and >40 years old on 7 May 2021) during the period when second doses were being given may have led to the exclusion of higher risk individuals from second dose ChAdOx1. Alternatively, those experiencing first dose adverse effects may not have proceeded with a second dose of ChAdOx1. Further work is required to understand if these reports or restrictions, or first dose adverse events had any role in the lower incidence we observed for second dose adverse events. We only included cases who received both vaccine doses. This will potentially oversample events occurring post-dose two, and hence may have tended to inflate the IRRs. We also assumed that the events of interest did not cause early deaths and thus censored the observation period. There were no deaths 0–27 days post-second dose ITP and CVST. The scenario of CVST being observed in the clearance period prior to second dose ChAdOx1 is possible. This is because CVST during the clearance period will not be due to the first dose ChAdOx1 vaccination (due to the gap in vaccination doses) and vaccination is recommended for individuals, even with past clotting episodes, as they remain at risk of COVID-19 disease^22^.

The increased risk found means further monitoring of CVST and ITP amongst those receiving second doses of adenovirus-based SARS-CoV-2 vaccines is warranted. Replication of our study in other countries is needed to confirm our results. We plan to update our analysis for booster doses and mRNA-1273 vaccines.

In conclusion, second dose BNT162b2 vaccination was not found to be associated with increased risks of thromboembolic, hemorrhagic or thrombocytopenic events. Second dose ChAdOx1 vaccination was associated with borderline increased risk of ITP and CVST events, however these events were rare and usually short-lived, thus providing reassurance about the safety of this vaccine. Further analyses including more cases are required to determine more precisely the risk profile for ITP and CVST after a second dose of ChAdOx1 vaccine.

## Methods

Ethical permission for this study was granted from South-East Scotland Research Ethics Committee 02 [12/SS/0201]. The Public Benefit and Privacy Panel Committee of Public Health Scotland approved the linkage and analysis of the de-identified datasets for this project [1920-0279].

### Study setting and population

The National Health Service in Scotland (NHS Scotland) provides comprehensive health services that are free at the point-of-care for all residents. Our base population for this study was 3.6 m (78.2% of eligible population) registered with a general medical practice (GP) in Scotland who had received a second dose of either ChAdOx1 or BNT162b2 vaccine. The baseline period comprised the remaining time from 1 December 2020 to 7 November 2021 or date of death, whichever came first, excluding clearance periods and risk periods (number of person-days).

### Study design

Following a pre-specified analysis plan, we carried out a self-controlled case series (SCCS) using the EAVE II prospective cohort. The GP clinical data contained records of the specified safety outcomes from 1 September 2019 through to 7 November 2021. A case was defined as anyone with a clinician recorded incident event of thrombocytopenia, venous thromboembolism, arterial thromboembolism or hemorrhage following the start of the COVID-19 vaccination program in Scotland. An incident case was defined as the first event in the 28-day period after a second dose.

### Data sources

Almost all residents in Scotland are registered with a GP and have a unique Community Health Index (CHI) number used by NHS Scotland. We used the CHI number to deterministically link all datasets with vaccination records in Public Health Scotland (PHS) (Extended Data Fig. 2). Vaccination information was extracted from the GP records and the Turas Vaccination Management Tool (TVMT) system; together these captured all vaccination records, including those vaccinated in general practices, community vaccination hubs and other settings such as care homes and hospitals in Scotland^23^. Further details on the data sources used in this study are available in a published project protocol^18^.

### Exposure definition

We studied second doses of the BNT162b2^24^ and ChAdOx1 vaccines^25^. We did not report Moderna second dose results due to the small number given in Scotland at the time of analysis. An individual was defined as exposed to each vaccine if they received their second dose vaccine from 11 January 2021.

### Outcomes

The outcomes analyzed in this study were one or more of: (i) ITP and other thrombocytopenic; (ii) venous or arterial thromboembolic; or (iii) hemorrhagic (excluding traumatic, gastrointestinal and genitourinary bleeding) events. We undertook additional a priori subgroup analyses focused on ITP, CVST, deep vein thrombosis (DVT) and pulmonary embolism (PE)^26^. Read codes (Version 2) were used to determine adverse incident events recorded in the primary care EHR data (Supplementary Table 1), which were then followed up for whether hospitalizations or deaths occurred in the linked Rapid Preliminary Inpatient Data (RAPID) dataset and National Records Scotland for hospitalization and mortality outcomes, respectively. The code lists used in this study were drawn up by the EAVE II clinical team and validated by experts in neurology and hematology. Incidence was the outcome of interest during the post vaccine exposure periods after vaccination; the primary incidence covered the 28 day period post-second dose and is expressed as number of events per 100,000 vaccinated.

### Statistical analysis

Incidence rates of the outcomes in the different exposure periods were calculated as the number of events in the exposure period divided by the person time in the exposure period. The SCCS models were fitted using a conditional Poisson regression model with an offset for the length of the risk period^27^. Incidence rate ratios (IRR) for each outcome of interest comparing risk periods relative to baseline periods (Fig. 1) were estimated by the SCCS model adjusting for week number as time-varying covariates. Exposure terms for each vaccine were included in the same model.

People were eligible for each study cohort if they had received at least two vaccine doses (either ChAdOx1 and BNT162b2), were at least 16 years old, and had a recording in the GP electronic health record (EHR) for the outcome of interest from the beginning of patient follow-up (i.e., 1 December 2020) to the earliest of the end of the study period (i.e., 7 November 2021) or when they died. If a patient had more than one adverse event of interest, only the first one was used.

The exposure variable was a second dose of the ChAdOx1 or BNT162b2 vaccines. We defined the exposure risk intervals as 0–6, 7–13, 14–20, and 21–27 days after the date of second vaccine dose. A 14-day clearance period was used prior to the exposure date (Fig. 1). To avoid overlapping risk periods, we assumed that later exposures (second dose) took precedence over the earlier one (first dose), except for the 14-day clearance period for the second dose.

Platelet counts for individuals were extracted from the GP EHR data. We extracted prescriptions related to ITP therapy and which may cause thrombocytopenia (Supplementary Table 2). These were used to determine duration of disease.

Analyses were carried out by one statistician (J.P.) and independently checked by an additional statistician (C.R.). All analyses were carried out with R software, version 3.6.1^28^.

### Reporting summary

Further information on research design is available in the Nature Research Reporting Summary linked to this article.

## Supplementary information

Supplementary Information

Peer Review File

Reporting Summary

## Data Availability

A data-dictionary covering the datasets used in this study can be found at https://github.com/EAVE-II/EAVE-II-data-dictionary. Patient-level data underlying this article is controlled and cannot be shared publicly due to data protection and confidentiality requirements. Data can be made available to approved researchers for analysis after securing relevant permissions from the data holders via the Public Benefit and Privacy Panel (PBPP). Enquiries regarding data availability should be directed to phs.edris@phs.scot. The normal expectation is that the application process will be concluded within 30 working days of submission, not including any time that has elapsed while waiting for a response.

## Code availability

All code used in this study is publicly available at https://github.com/EAVE-II/Covid-vaccine-safety-haemo.
