# Supplementary material for: Second-dose ChAdOx1 and BNT162b2 COVID-19 vaccines and thrombocytopenic, thromboembolic and hemorrhagic events in Scotland
Source: Nat Commun. 2022 Aug 15;13:4800. doi: 10.1038/s41467-022-32264-6 (PMC9377297; doi:10.1038/s41467-022-32264-6)

Supplementary File

Table 1. Read codes for outcomes of interest

Code	Description
**Thrombocytopenia**	
42P2	Thrombocytopenia
D313.	Primary thrombocytopenia
D3133	[X]Essential thrombocytopenia NOS
D313y	Other specified primary thrombocytopenia
D313z	Primary thrombocytopenia NOS
D314	Secondary thrombocytopenia
D3141	Thrombocytopenia due to drugs
D314y	Other specified secondary thrombocytopenia
D314z	Secondary thrombocytopenia NOS
D315	Thrombocytopenia NOS
Dyu32	[X]Other primary thrombocytopenia
**Idiopathic thrombocytopenic purpura (ITP)**	
D3130	Idiopathic thrombocytopenic purpura
**Venous thromboembolic events**	
8CMWA	On deep vein thrombosis care pathway [s]
G801.	Deep vein phlebitis and thrombophlebitis of the leg [s]
G801B	Deep vein thrombophlebitis of the leg unspecified [s]
G801D	Deep vein thrombosis of lower limb [s]
G801F	Deep vein thrombosis of peroneal vein [s]
G801z	Deep vein phlebitis and thrombophlebitis of the leg NOS [s]
G8020	Thrombosis of vein of leg [s]
G80y4	Thrombophlebitis of the common iliac vein
G80y6	Thrombophlebitis of the external iliac vein

G401.	Pulmonary embolism [b]
G4011	Recurrent pulmonary embolism [b]
G81..	Portal vein thrombosis
G82..	Other venous embolism and thrombosis
G820.	Budd - Chiari syndrome (hepatic vein thrombosis)
G821.	Thrombophlebitis migrans
G822.	Embolism and thrombosis of the vena cava
G823.	Embolism and thrombosis of the renal vein
G82y.	Other embolism and thrombosis
G82z.	Embolism and thrombosis NOS
G82z0	Embolus of vein NOS
G82z1	Thrombosis of vein NOS
G82zz	Embolism and thrombosis NOS
F4238	Central retinal vein occlusion
8HTm.	Referral to deep vein thrombosis clinic
**Cerebral venous sinus thrombosis (CVST)**	
G67A.	Cerebral vein thrombosis
F051z	Thrombosis of central nervous system venous sinus NOS
F053.	Thrombophlebitis of central nervous system venous sinuses
G676.	Nonpyogenic venous sinus thrombosis
F05..	Phlebitis and thrombophlebitis of intracranial sinuses
F050.	Embolism of central nervous system venous sinus
F0500	Embolism cavernous sinus
F0501	Embolism superior longitudinal sinus
F0502	Embolism lateral sinus
F0503	Embolism transverse sinus
F050z	Embolism central nervous system venous sinus NOS
F051.	Thrombosis of central nervous system venous sinuses

F0510	Thrombosis cavernous sinus
F0511	Thrombosis of superior longitudinal sinus
F0512	Thrombosis lateral sinus
F0513	Thrombosis transverse sinus
F052.	Phlebitis of central nervous system venous sinuses
F0520	Phlebitis cavernous sinus
F0521	Phlebitis of superior longitudinal sinus
F0522	Phlebitis lateral sinus
F0523	Phlebitis transverse sinus
F052z	Phlebitis of central nervous system venous sinus NOS
F0530	Thrombophlebitis of cavernous sinus
F0531	Thrombophlebitis of superior longitudinal venous sinus
F0532	Thrombophlebitis lateral venous sinus
F053z	Thrombophlebitis of central nervous system venous sinus NOS
F05z.	Phlebitis or thrombophlebitis of CNS venous sinus NOS
**Arterial thromboembolic events**	
G63..	Precerebral arterial occlusion
G630.	Basilar artery occlusion
G631.	Carotid artery occlusion
G632.	Vertebral artery occlusion
G633.	Multiple and bilateral precerebral arterial occlusion
G634.	Carotid artery stenosis
G63y.	Other precerebral artery occlusion
G63y0	Cerebral infarct due to thrombosis of precerebral arteries
G63y1	Cerebral infarction due to embolism of precerebral arteries
G63z.	Precerebral artery occlusion NOS
G64..	Cerebral arterial occlusion
G640.	Cerebral thrombosis

G6400	Cerebral infarction due to thrombosis of cerebral arteries
G641.	Cerebral embolism
G6410	Cerebral infarction due to embolism of cerebral arteries
G64z.	Cerebral infarction NOS
G64z0	Brainstem infarction
G64z1	Wallenberg syndrome
G64z2	Left sided cerebral infarction
G64z3	Right sided cerebral infarction
G65..	Transient cerebral ischaemia
G650.	Basilar artery syndrome
G651.	Vertebral artery syndrome
G6510	Vertebro-basilar artery syndrome
G652.	Subclavian steal syndrome
G653.	Carotid artery syndrome hemispheric
G654.	Multiple and bilateral precerebral artery syndromes
G65y.	Other transient cerebral ischaemia
G65z.	Transient cerebral ischaemia NOS
G65z0	Impending cerebral ischaemia
G65z1	Intermittent cerebral ischaemia
G65zz	Transient cerebral ischaemia NOS
G66..	Stroke and cerebrovascular accident unspecified
G660.	Middle cerebral artery syndrome
G661.	Anterior cerebral artery syndrome
G662.	Posterior cerebral artery syndrome
G663.	Brain stem stroke syndrome
G664.	Cerebellar stroke syndrome
G665.	Pure motor lacunar syndrome
G666.	Pure sensory lacunar syndrome

G667.	Left sided CVA
G668.	Right sided CVA
G67..	Other cerebrovascular disease
G670.	Cerebral atherosclerosis
G671.	Generalised ischaemic cerebrovascular disease NOS
G6710	Acute cerebrovascular insufficiency NOS
G6711	Chronic cerebral ischaemia
G671z	Generalised ischaemic cerebrovascular disease NOS
G6730	Dissection of cerebral arteries, nonruptured
G677.	Occlusion/stenosis cerebral arts not result cerebral infarct
G6770	Occlusion and stenosis of middle cerebral artery
G6771	Occlusion and stenosis of anterior cerebral artery
G6772	Occlusion and stenosis of posterior cerebral artery
G6773	Occlusion and stenosis of cerebellar arteries
G6774	Occlusion+stenosis of multiple and bilat cerebral arteries
G67y.	Other cerebrovascular disease OS
G67z.	Other cerebrovascular disease NOS
G68..	Late effects of cerebrovascular disease
G680.	Sequelae of subarachnoid haemorrhage
G681.	Sequelae of intracerebral haemorrhage
G682.	Sequelae of other nontraumatic intracranial haemorrhage
G683.	Sequelae of cerebral infarction
G68W.	Sequelae/other + unspecified cerebrovascular diseases
G68X.	Sequelae of stroke,not specfd as h'morrhage or infarction
G6W..	Cereb infarct due unsp occlus/stenos precerebr arteries
G6X..	Cerebrl infarctn due/unspcf occlusn or sten/cerebrl artrs
G6y..	Other specified cerebrovascular disease
G74..	Arterial embolism and thrombosis

G740.	Embolism and thrombosis of the abdominal aorta
G741.	Embolism and thrombosis of the thoracic aorta
G742.	Embolism and thrombosis of an arm or leg artery
G7420	Embolism and thrombosis of the brachial artery
G7421	Embolism and thrombosis of the radial artery
G7422	Embolism and thrombosis of the ulnar artery
G7423	Embolism and thrombosis of an arm artery NOS
G7424	Embolism and thrombosis of the femoral artery
G7425	Embolism and thrombosis of the popliteal artery
G7426	Embolism and thrombosis of the anterior tibial artery
G7427	Embolism and thrombosis of the dorsalis pedis artery
G7428	Embolism and thrombosis of the posterior tibial artery
G7429	Embolism and thrombosis of a leg artery NOS
G742z	Peripheral arterial embolism and thrombosis NOS
G743.	Embolism and thrombosis of other and unspec parts aorta
G74y.	Embolism and thrombosis of other specified artery
G74y0	Embolism and/or thrombosis of the common iliac artery
G74y1	Embolism and/or thrombosis of the internal iliac artery
G74y2	Embolism and/or thrombosis of the external iliac artery
G74y3	Embolism and thrombosis of the iliac artery unspecified
G74y5	Embolism and thrombosis of the subclavian artery
G74y6	Embolism and thrombosis of the splenic artery
G74y7	Embolism and thrombosis of the axillary artery
G74y8	Embolism and thrombosis of the coeliac artery
G74y9	Embolism and thrombosis of the hepatic artery
G74yz	Embolism and thrombosis of other arteries NOS
G74z.	Arterial embolism and thrombosis NOS
F423.	Retinal vascular occlusion

F4230	Unspecified retinal vascular occlusion
F4231	Central retinal artery occlusion
F4232	Retinal arterial branch occlusion
F4233	Retinal microembolism
F4234	Hollenhorst plaque
F4235	Retinal partial arterial occlusion NOS
F4236	Amaurosis fugax
F4237	Retinal transient arterial occlusion NOS
G3...	Ischaemic heart disease
G30..	Acute myocardial infarction
G300.	Acute anterolateral infarction
G301.	Other specified anterior myocardial infarction
G3010	Acute anteroapical infarction
G3011	Acute anteroseptal infarction
G301z	Anterior myocardial infarction NOS
G302.	Acute inferolateral infarction
G303.	Acute inferoposterior infarction
G304.	Posterior myocardial infarction NOS
G305.	Lateral myocardial infarction NOS
G306.	True posterior myocardial infarction
G307.	Acute subendocardial infarction
G3070	Acute non-Q wave infarction
G3071	Acute non-ST segment elevation myocardial infarction
G308.	Inferior myocardial infarction NOS
G309.	Acute Q-wave infarct
G30B.	Acute posterolateral myocardial infarction
G30X.	Acute transmural myocardial infarction of unspecif site
G30X0	Acute ST segment elevation myocardial infarction

G30y.	Other acute myocardial infarction
G30y0	Acute atrial infarction
G30y1	Acute papillary muscle infarction
G30y2	Acute septal infarction
G30yz	Other acute myocardial infarction NOS
G30z.	Acute myocardial infarction NOS
G31..	Other acute and subacute ischaemic heart disease
G310.	Postmyocardial infarction syndrome
G310.	Dressler's syndrome
G3...	Arteriosclerotic heart disease
G311.	Preinfarction syndrome
G3110	Myocardial infarction aborted
G3111	Unstable angina
G3112	Angina at rest
G3113	Refractory angina
G3114	Worsening angina
G3115	Acute coronary syndrome
G311z	Preinfarction syndrome NOS
G312.	Coronary thrombosis not resulting in myocardial infarction
G31y.	Other acute and subacute ischaemic heart disease
G31y0	Acute coronary insufficiency
G31y1	Microinfarction of heart
G31y2	Subendocardial ischaemia
G31y3	Transient myocardial ischaemia
G31yz	Other acute and subacute ischaemic heart disease NOS
G35..	Subsequent myocardial infarction
G350.	Subsequent myocardial infarction of anterior wall
G351.	Subsequent myocardial infarction of inferior wall

G353.	Subsequent myocardial infarction of other sites
G35X.	Subsequent myocardial infarction of unspecified site
G36..	Certain current complication follow acute myocardial infarct
G360.	Haemopericardium/current comp folow acut myocard infarct
G361.	Atrial septal defect/curr comp folow acut myocardal infarct
G362.	Ventric septal defect/curr comp fol acut myocardal infarctn
G363.	Ruptur cardiac wall w'out haemopericard/cur comp fol ac MI
G364.	Ruptur chordae tendinae/curr comp fol acute myocard infarct
G365.	Rupture papillary muscle/curr comp fol acute myocard infarct
G38..	Postoperative myocardial infarction
G380.	Postoperative transmural myocardial infarction anterior wall
G381.	Postoperative transmural myocardial infarction inferior wall
G384.	Postoperative subendocardial myocardial infarction
G38z.	Postoperative myocardial infarction; unspecified
G3y..	Other specified ischaemic heart disease
G3z..	Ischaemic heart disease NOS
Gyu32	[X]Other forms of acute ischaemic heart disease
Gyu34	[X]Acute transmural myocardial infarction of unspecif site
Gyu36	[X]Subsequent myocardial infarction of unspecified site
**Haemorrhage**	
G61..	Intracerebral haemorrhage
G610.	Cortical haemorrhage
G611.	Internal capsule haemorrhage
G612.	Basal nucleus haemorrhage
G613.	Cerebellar haemorrhage
G614.	Pontine haemorrhage
G615.	Bulbar haemorrhage
G617.	Intracerebral haemorrhage, intraventricular

G619.	Lobar cerebral haemorrhage
G61X.	Intracerebral haemorrhage in hemisphere, unspecified
G61X0	Left sided intracerebral haemorrhage, unspecified
G61X1	Right sided intracerebral haemorrhage, unspecified
G61z.	Intracerebral haemorrhage NOS
G62..	Other and unspecified intracranial haemorrhage
G620.	Extradural haemorrhage – non-traumatic
G621.	Subdural haemorrhage – non-traumatic
G623.	Subdural haemorrhage NOS
G62z.	Intracranial haemorrhage NOS
2BB5.	O/E - retinal haemorrhages
2BB8.	O/E - vitreous haemorrhages
2DE7.	O/E - throat haemorrhage
C1542	Adrenal haemorrhage
F4045	Intraocular haemorrhage
F42y1	Superficial retinal haemorrhage
F42y3	Deep retinal haemorrhage
F42y4	Subretinal haemorrhage
F42y5	Retinal haemorrhage NOS
F436.	Choroidal haemorrhage and rupture
F4360	Unspecified choroidal haemorrhage
F4361	Expulsive choroidal haemorrhage
F436z	Choroidal haemorrhage or rupture NOS
F4C72	Conjunctival haemorrhage NOS
F4Ey0	Haemorrhage of eyelid
F4H41	Optic nerve sheath haemorrhage
F4K28	Vitreous haemorrhage
F4K7.	Retrobulbar haemorrhage

FyuH4	[X]Vitreous haemorrhage in diseases classified elsewhere
G8y0.	Haemorrhage NOS
Gyu61	[X]Other subarachnoid haemorrhage
Gyu62	[X]Other intracerebral haemorrhage
Gyu6F	[X]Intracerebral haemorrhage in hemisphere, unspecified
K1381	Renal artery haemorrhage
R048.	[D]Throat haemorrhage
R0631	[D]Pulmonary haemorrhage NOS
R09z0	[D]Umbilical bleeding
G60..	Subarachnoid haemorrhage
G601.	Subarachnoid haemorrhage from carotid siphon and bifurcation
G602.	Subarachnoid haemorrhage from middle cerebral artery
G603.	Subarachnoid haemorrhage from anterior communicating artery
G604.	Subarachnoid haemorrhage from posterior communicating artery
G605.	Subarachnoid haemorrhage from basilar artery
G60z.	Subarachnoid haemorrhage NOS
D3...	Clotting and bleeding disorders
D30..	Coagulation defects
D3z..	Clotting or bleeding disorder NOS

[a] Deep vein thrombosis [b] Pulmonary embolism

Table 2. Prescriptions related to idiopathic thrombocytopenic purpura

	**Drug name**
ITP causing prescriptions	Glycoprotein IIb/IIIa inhibitors (tirofiban, abciximab, and eptifibatide), heparin, cephalosporins, linezolid, penicillins, sulphonamides, trimethoprim, vancomycin, quinine, ethambutol, rifampicin, carbamazepine, phenytoin, sodium valproate, ibuprofen, naproxen, abciximab, amiodarone, eptifibatide, furosemide, quinidine, thiazides, tirofiban, haloperidol, paracetamol, irinotecan, mirtazapine, oxaliplatin, salmeterol, tacrolimus and ciprofloxacin
ITP therapy	Azathioprine, ciclosporin, cyclophosphamide, danazol, dapsone, intravenous immunoglobulin, mycophenolate, oral corticosteroids, rituximab, vinca alkaloids, eltrombopag, and romiplostim therapy

ITP: idiopathic thrombocytopenic purpura

Table S3. Reporting STROBE and RECORD checklists

	Item No.	STROBE items	RECORD items	Location in manuscript where items are reported
Title and abstract				
	1	(a) Indicate the study's design with a commonly used term in the title or the abstract (b) Provide in the abstract an informative and balanced summary of what was done and what was found	RECORD 1.1: The type of data used should be specified in the title or abstract. When possible, the name of the databases used should be included.<br><br>RECORD 1.2: If applicable, the geographic region and timeframe within which the study took place should be reported in the title or abstract.<br><br>RECORD 1.3: If linkage between databases was conducted for the study, this should be clearly stated in the title or abstract.	p. 1-2
Introduction				
Background rationale	2	Explain the scientific background and rationale for the investigation being reported		p. 3
Objectives	3	State specific objectives, including any prespecified hypotheses		p. 3
Methods				
Study Design	4	Present key elements of study design early in the paper		p. 10

Setting	5	Describe the setting, locations, and relevant dates, including periods of recruitment, exposure, follow-up, and data collection		p. 10
Participants	6	*(a) Cohort study* - Give the eligibility criteria, and the sources and methods of selection of participants. Describe methods of follow-up<br><br>*Case-control study* - Give the eligibility criteria, and the sources and methods of case ascertainment and control selection. Give the rationale for the choice of cases and controls<br><br>*Cross-sectional study* - Give the eligibility criteria, and the sources and methods of selection of participants<br><br>*(b) Cohort study* - For matched studies, give matching criteria and number of exposed and unexposed<br><br>*Case-control study* - For matched studies, give matching criteria and the number of controls per case	RECORD 6.1: The methods of study population selection (such as codes or algorithms used to identify subjects) should be listed in detail. If this is not possible, an explanation should be provided.<br><br>RECORD 6.2: Any validation studies of the codes or algorithms used to select the population should be referenced. If validation was conducted for this study and not published elsewhere, detailed methods and results should be provided.<br><br>RECORD 6.3: If the study involved linkage of databases, consider use of a flow diagram or other graphical display to demonstrate the data linkage process, including the number of individuals with linked data at each stage.	p. 10
Variables	7	Clearly define all outcomes, exposures, predictors, potential confounders, and effect modifiers. Give diagnostic criteria, if applicable.	RECORD 7.1: A complete list of codes and algorithms used to classify exposures, outcomes, confounders, and effect modifiers should be provided. If these cannot be reported, an explanation should be provided.	p. 11

Data sources/ measurement	8	For each variable of interest, give sources of data and details of methods of assessment (measurement).<br>Describe comparability of assessment methods if there is more than one group		p. 11-12
Bias	9	Describe any efforts to address potential sources of bias		p. 12
Study size	10	Explain how the study size was arrived at		N/A
Quantitative variables	11	Explain how quantitative variables were handled in the analyses. If applicable, describe which groupings were chosen, and why		p. 12
Statistical methods	12	(a) Describe all statistical methods, including those used to control for confounding<br>(b) Describe any methods used to examine subgroups and interactions<br>(c) Explain how missing data were addressed<br>(d) *Cohort study* - If applicable, explain how loss to follow-up was addressed<br>*Case-control study* - If applicable, explain how matching of cases and controls was addressed<br>*Cross-sectional study* - If applicable, describe analytical methods taking account of sampling strategy<br>(e) Describe any sensitivity analyses		p. 12

Data access and cleaning methods		..	RECORD 12.1: Authors should describe the extent to which the investigators had access to the database population used to create the study population.<br><br>RECORD 12.2: Authors should provide information on the data cleaning methods used in the study.	N/A
Linkage		..	RECORD 12.3: State whether the study included person-level, institutional-level, or other data linkage across two or more databases. The methods of linkage and methods of linkage quality evaluation should be provided.	p. 12
Results				
Participants	13	(a) Report the numbers of individuals at each stage of the study (*e.g.*, numbers potentially eligible, examined for eligibility, confirmed eligible, included in the study, completing follow-up, and analysed)<br><br>(b) Give reasons for non-participation at each stage.<br><br>(c) Consider use of a flow diagram	RECORD 13.1: Describe in detail the selection of the persons included in the study (*i.e.,* study population selection) including filtering based on data quality, data availability and linkage. The selection of included persons can be described in the text and/or by means of the study flow diagram.	p. 4

Descriptive data	14	(a) Give characteristics of study participants (*e.g.*, demographic, clinical, social) and information on exposures and potential confounders<br><br>(b) Indicate the number of participants with missing data for each variable of interest<br><br>(c) *Cohort study* - summarise follow-up time (*e.g.*, average and total amount)		p. 4
Outcome data	15	*Cohort study* - Report numbers of outcome events or summary measures over time<br><br>*Case-control study* - Report numbers in each exposure category, or summary measures of exposure<br><br>*Cross-sectional study* - Report numbers of outcome events or summary measures		p. 4-6
Main results	16	(a) Give unadjusted estimates and, if applicable, confounder-adjusted estimates and their precision (e.g., 95% confidence interval). Make clear which confounders were adjusted for and why they were included<br><br>(b) Report category boundaries when continuous variables were categorized<br><br>(c) If relevant, consider translating estimates of relative risk into absolute risk for a meaningful time period		p. 4-6

Other analyses	17	Report other analyses done—e.g., analyses of subgroups and interactions, and sensitivity analyses		p. 4-6
Discussion				
Key results	18	Summarise key results with reference to study objectives		p. 6-7
Limitations	19	Discuss limitations of the study, taking into account sources of potential bias or imprecision. Discuss both direction and magnitude of any potential bias	RECORD 19.1: Discuss the implications of using data that were not created or collected to answer the specific research question(s). Include discussion of misclassification bias, unmeasured confounding, missing data, and changing eligibility over time, as they pertain to the study being reported.	p. 7-8
Interpretation	20	Give a cautious overall interpretation of results considering objectives, limitations, multiplicity of analyses, results from similar studies, and other relevant evidence		p. 9
Generalisability	21	Discuss the generalisability (external validity) of the study results		p.9
Other Information				
Funding	22	Give the source of funding and the role of the funders for the present study and, if applicable, for the original study on which the present article is based		p. 16

Accessibility of protocol, raw data, and programming code		..	RECORD 22.1: Authors should provide information on how to access any supplemental information such as the study protocol, raw data, or programming code.	p. 12

Figure 1. Second dose vaccine uptake by type of vaccine for individuals in Scotland, up to 7 November 2021: (a) BNT162b2 and (b) ChAdOx1

(a)

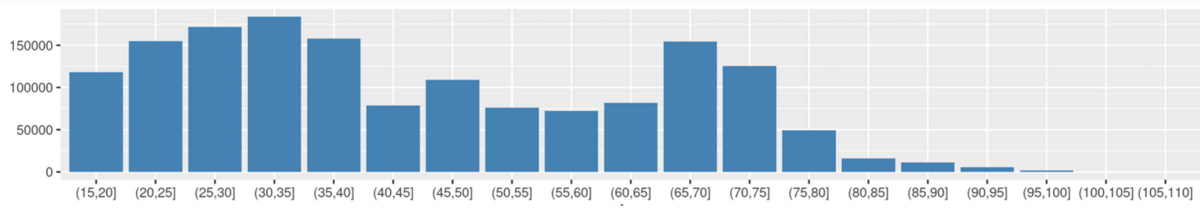

(b)

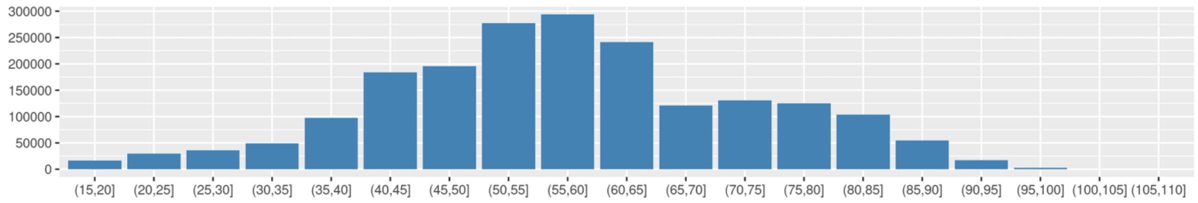

Figure 2. Data linkage diagram

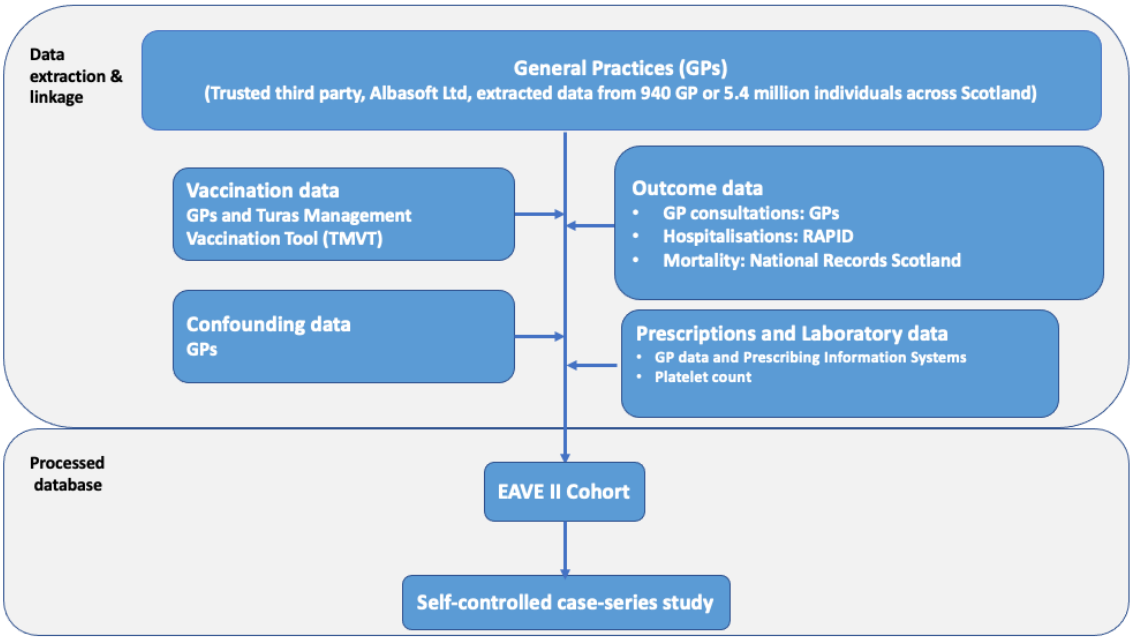

Note: Community Health Index (CHI) numbers were used to link all datasets. Details on these datasets are available in our published protocol (Simpson CR, Robertson C, Vasileiou E, et al. Early Pandemic Evaluation and Enhanced Surveillance of COVID-19 (EAVE II): protocol for an observational study using linked Scottish national data. BMJ Open 2020;10:e039097. doi: 10.1136/bmjopen-2020-039097) There are two core methods of recording vaccine delivery, the national Turas Management Vaccination Tool (TMVT) system and local GP IT systems. TMVT was developed as a web application by National Education Scotland (NES). It is in general the preferred method of recording a vaccination where this is done outside the normal vaccine locations, predominantly dedicated vaccination centres and community programmes. Most vaccinations delivered in general practice settings are recorded in local IT systems; there are a few geographical areas that have however mandated the use of TMVT in every setting, including GP practices. Currently GP's are paid per 100 vaccines administered so they are highly motivated to record information accurately. If this is not recorded to a minimum standard, they will not receive payment. All vaccines administered through vaccination centres and community programmes are accounted for on a daily basis. All vaccines recorded via TMVT are transferred to the national clinical datastore (NCDS) then to Albasoft on a daily basis. At 9pm each night, these are loaded into a secure database and each practice "polls" the data store as part of the ESCRO data pump run between 12:00am and 5:00am each day to request the records for their specific practice. These are then loaded into a local queue at the practice for processing later in the day. As part of the same data pump run, the local GP IT system is queried and all vaccination records for the previous day are extracted (with a 10 day overlap to catch any retrospective recording) These records are then transferred back to Albasoft and collated into a single data source which is returned to the National Clinical Data Store (NCDS) at 8am each morning. As a result, all vaccinations recorded either by TMVT or GP IT systems pass through Albasoft in a 24-hour cycle. As part of the agreement to provide these data for EAVE II, vaccination records from both the TMVT and GP IT systems are transferred each day following the National Clinical Data Store processing to the EAVE II secure datastore in Public Health Scotland (PHS). This ensures that the EAVE II data are as up to date as possible. It is therefore extremely unlikely that any vaccinations will have been missed.

Figure 3. Days interval between first and second dose vaccine by type of vaccine for individuals in Scotland, up to 7 November 2021: (a) BNT162b2 and (b) ChAdOx1

(a)

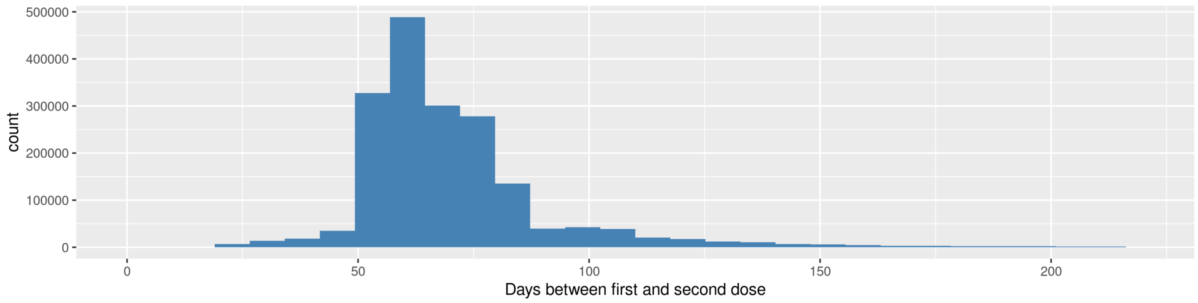

(b)

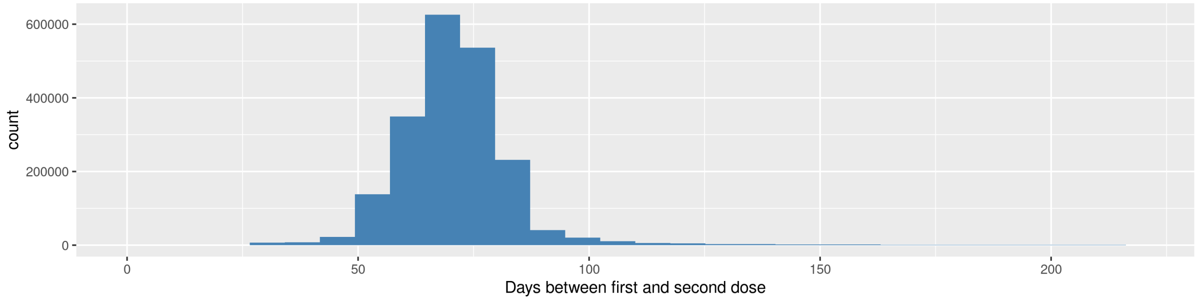

Supplement: Supplementary file 1 — Supplementary Information [file 41467_2022_32264_MOESM1_ESM.pdf]
