# Supplementary material for: Second-dose ChAdOx1 and BNT162b2 COVID-19 vaccines and thrombocytopenic, thromboembolic and hemorrhagic events in Scotland
Source: Nat Commun. 2022 Aug 15;13:4800. doi: 10.1038/s41467-022-32264-6 (PMC9377297; doi:10.1038/s41467-022-32264-6)
Supplement: Supplementary file 2 — Peer Review File [file 41467_2022_32264_MOESM2_ESM.pdf]

nature portfolio

Peer Review File

Second-dose ChAdOx1 and BNT162b2 COVID-19 vaccines and thrombocytopenic, thromboembolic and hemorrhagic events in Scotland

Open Access This file is licensed under a Creative Commons Attribution 4.0 International License, which permits use, sharing, adaptation, distribution and reproduction in any medium or format, as long as you give appropriate credit to the original author(s) and the source, provide a link to the Creative Commons license, and indicate if changes were made. In the cases where the authors are anonymous, such as is the case for the reports of anonymous peer reviewers, author attribution should be to 'Anonymous Referee' followed by a clear attribution to the source work. The images or other third party material in this file are included in the article's Creative Commons license, unless indicated otherwise in a credit line to the material. If material is not included in the article's Creative Commons license and your intended use is not permitted by statutory regulation or exceeds the permitted use, you will need to obtain permission directly from the copyright holder. To view a copy of this license, visit http://creativecommons.org/licenses/by/4.0/.

Reviewers' Comments:

Reviewer #1:
Remarks to the Author:
This study provides evidence of safety of ChAdOx1 and BNT162b2 vaccines in relation to thromboembolic and haemorrhagic adverse events within 4 weeks of the second vaccine dose. The authors suggest there may be an increased risk of ITP and CVST following second doses of the ChAdOx1 but not the BNT162b2 vaccines. In my view, the numbers of events for these latter endpoints are too small to draw firm conclusions. Overall, the study provides reassurance about the safety of these vaccines, but points to the need for further work to determine their risk profile with greater accuracy.

1. Assessment of positive and negative findings

I think that the findings relating to CVST, and to a lesser extent ITP, as presented at lines 169-170 and in the Abstract, are overstated. The data are very sparse, and hence IRR estimates for sub-intervals of the 0-27 day risk period lack robustness. Thus, from Table 1, an IRR of 5.01 95% CI (1.16, 21.69) is obtained for the 7-13 day post-vaccination period, yet the IRR for the whole 0-27 day risk period is 1.00 (0.20, 4.92), indicating no increase at all. Unless there are strong a priori reasons to focus on the 7-13 day sub-interval, this contrast suggests lack of robustness, or less compellingly that the vaccine alters the timing of events within the interval. In addition, the number of events in the 7-13 day risk period being ≤ 5 (perhaps only 2 or 3), the asymptotic assumptions upon which the calculation of the confidence interval is based are of questionable validity. This is especially so in view of the (presumably) comparatively large number of parameters being estimated.

A more accurate description of the results, in my view, would reflect the tentative nature of the evidence and might go something like this: "No evidence of an increased risk was found for the 0-27 day risk period (IRR 1.00, 95% CI 0-20 to 4.92). However, few (≤ 5) events arose within this risk period. It is perhaps noteworthy that these events all clustered in the 7-13 day period. Further analyses including more cases are required to determine more precisely the risk profile for CVST after a second dose of ChAdOx1 vaccine".

Likewise, the authors choose to highlight a "borderline increased risk" of ITP after second dose of ChAdOx1 vaccine, but only in the 14-20 day period. The IRR for the 0-27 day risk period as a whole is 1.16 (0.62, 2.17), which is not suggestive of a real effect; 6 of the 13 events in this risk period arose in the 14-20 day sub-interval. The IRRs suggest that the distribution of the 13 events in the risk period might be 2,3,6,2, which hardly seems like compelling evidence of clustering: unless there is powerful a priori evidence to think that ITP might be clustered 14-20 days after vaccination, this distribution could just as well be a chance effect. At most, the results might suggest that further investigation in more powerful studies should be undertaken.

On the other hand, it is perhaps noteworthy that 6/8 IRRs for the 0-27 day risk period for ChAdOx1 vaccine and 7/8 for BNT162b2 are less than 1. Several of these IRRs suggest a statistically significantly protective effect. For ChAdOx1 vaccine, this is the case for VTE, PE, ATE, and haemorrhagic events; and for BNT262b2, for VTE, DVT, PE, and ATE. This is most likely due to well-vaccinee bias, as people who are unwell defer vaccination. Alternatively, it might conceivably be a by-product of vaccine protection against COVID-19, if COVID is a cause of these events (I presume that COVID infections were not allowed for in the analysis as time-varying confounders). Of all the findings of the paper, these are the most robust. Might they not be worthy of at least brief comment in the Discussion?

2. SCCS assumptions

The methods seem generally sound but would benefit from discussion of the validity of the two key

assumptions.

The first assumption is that occurrence of an event does not lead to the cancellation of vaccination. If such cancellations do in fact occur, then including only cases who have received both vaccine doses, as done here, will oversample events occurring post dose 2, and hence may tend to inflate the IRRs. Since no compellingly positive associations are found, this is not a major issue. (A simpler analysis with observation periods starting at second dose would avoid the issue entirely.)

The second assumption is that the events of interest do not cause early deaths and thus censor the observation period (deaths from other causes are not at issue). Some comment on this would be appropriate, including mention of the numbers of cases and proportions who died during the observation period.

3. Data consistency

The data description and data tabulations sometimes appear inconsistent. For example for CVST and BNT262b2: according to Table 2 there are 9 events in a baseline period. If each of these corresponds to a separate case (as stated on line 333) and all cases receive a second dose (as stated on line 328) then all these 9 cases should have at least one day of exposure to a second dose. Yet the person-time presented in Table 2 suggests that only 6 cases are left at this point.

4. Further points

The following points came to mind while reading the paper.

Line 140: “this is one of the first real-world contemporaneous studies”: another is Jabagi et al (2022), Myocardial infarction, stroke, and pulmonary embolism after BNT162b2 COVID-19 vaccine in people aged 75 years or older. JAMA 2022; 327(1):80-82. Doi:10.1001/jama.2021.21699.

Lines 151-2 Possible shortcomings of the READ codes: is anything known about the positive predictive values of these codes for the events studied? This is relevant because low PPV will bias results towards the null. Also, how accurate are the event timings? This is particularly relevant to the interpretation of timings of events within the 0-27 day risk period.

Tables 1 and 2: please clarify whether the data given for the baseline and clearance periods relate to all 3 baseline and both clearance periods indicated in Figure 1, or just those adjacent to the dose 2 risk period.

Tables 1 and 2: The CIs quoted when there are 0 events are (0.00, Inf): this is not strictly correct. When there are 0 events, the CI cannot be calculated using conditional Poisson regression (though other methods could be used), so strictly speaking no CI should be quoted.

Paddy Farrington
The Open University, UK

Reviewer #2:
Remarks to the Author:
This is a brief report on the risk of thromboembolic and thrombocytopenic adverse events following second doses of AZ and BNT in a nationwide Scottish population. The authors utilize a SCCS approach and observe an association between AZ and CVST in the 7 to 13 days period following the second dose. I would like the authors to elaborate on the following issues.

1. The VITT signal was identified in younger females. This study does not really evaluate the risk of a second dose of AZ in younger females. Please discuss the relevance of these results to the VITT signal in more detail. The authors found 0 cases of CVST in <30 yr olds. Is it possible to evaluate how strong a signal could have been detected given the number of <30 yr old females in Scotland with a second dose of AZ?
2. Please address multiple comparisons. The authors have conducted a large number of comparisons including using multiple risk periods. Would it not have made more sense to pre-specify a main risk period based on the VITT signal, alternatively a 28-day period? In the 28-day period following AZ2 the CVST association was 1.00 (0.20-4.92). The 7-13 day association could be a random finding.
3. Discuss why a CVST association is observed in the clearance period. This is puzzling to me. Who would receive a CVST diagnosis and go on to be vaccinated in the next 2 weeks.
4. What is the distribution of the dose-interval between AZ1 and AZ2 in the Scottish setting?
5. Please argue (using sensitivity analyses) that the assumptions of the SCCS approach is fulfilled. E.g. is it likely that an outcome event does not influence future vaccination propensity?
6. The authors use READ codes from the GP setting (as I understand the manuscript) with follow up in hospital data. Would it not make more sense to use the hospital data to identify something like CVST or pulmonary embolism? Presumably cases are missed by using GP data?
7. Have these READ codes been validated for thromboembolic- and thrombocytopenic outcomes?
8. How was incidences calculated? This is not described in the Methods.
9. Discuss these incidences in contrast to what has been observed after AZ1.
10. L340-342: I did not understand how this was done or what it was used for.

Reviewer #3:
Remarks to the Author:

The study reports on the incidence of adverse events after the second dose of COVID-19 vaccines. The data are of interest but the limited information on the details of the few patients who developed these adverse events make it very difficult to draw further conclusions.
Page 2, line 32-32: Did the individuals receive both ChAdOx1 as first and BNT162b2 as second dose, as implied by "and"? Probably "or" would be more clear.
Page 3, line 56: Refs 6, 7, 8 do not report on ITP but on VITT
Page 4. Line 79: please explain IRR
Abstract, line 34-35 states that ChAdOx1 is associated with a borderline increased risk for ITP, Page 4, line 78 states that there is no increased risk for thrombocytopenia. This is confusing.
Page 5, line 93-94: 1310 and 10077% are likely an error
Page 5, line 95-96: There are only two drugs, which induce ITP, i.e. gold and alemtuzumab. It would be very unusual if the patients did receive these two drugs. Please do not confuse ITP with drug dependent thrombocytopenia, which is a very different mechanism although both are antibody mediated.
Page 5, last para: this observation is highly interesting. CVST without thrombosis argues against VITT/TTS as underlying cause and hints towards the possibility that the vaccine itself is altering the cerebral veins. Any information on the platelet counts in these patients would be important, either known baseline platelet counts or a potential increase in platelet counts after treatment.
Are there any data on the incidence of VITT/TTS? At the time of the second dose anti-PF4 antibodies have widely been tested in patients with adverse events after vaccination.
Page 7, line 149-150: it is striking that adverse events after vaccination with ChAdOx1 in LIMCs are very rarely reported. It is still unclear whether this is related to underreporting or to different susceptibility. Therefore wording related to generalizability should be more careful

Point-by-point responses

Detailed point-by-point revisions are provided below in the order in which they were raised. For your convenience, we reproduce the reviewers feedback in full (in bold) before detailing our responses.

Reviewer #1:

1. This study provides evidence of safety of ChAdOx1 and BNT162b2 vaccines in relation to thromboembolic and haemorrhagic adverse events within 4 weeks of the second vaccine dose. The authors suggest there may be an increased risk of ITP and CVST following second doses of the ChAdOx1 but not the BNT162b2 vaccines. In my view, the numbers of events for these latter endpoints are too small to draw firm conclusions. Overall, the study provides reassurance about the safety of these vaccines, but points to the need for further work to determine their risk profile with greater accuracy.

Response: Thank you for these comments. We agree that the study provides reassurance about the safety of these vaccines. We have clarified these two points in our paper by amending our Discussion in the Abstract and Conclusion in our main text:

Abstract (Line 46) *"Second dose ChAdOx1 vaccination was associated with increased borderline risks of ITP and CVST events. However these events were rare thus providing reassurance about the safety of these vaccines. Further analyses including more cases are required to determine more precisely the risk profile for ITP and CVST after a second dose of ChAdOx1 vaccine."*

Conclusion (Line 259) *"Second dose ChAdOx1 vaccination was associated with borderline increased risk of ITP and CVST events, however these events were rare and usually short-lived, thus providing reassurance about the safety of this vaccine. Further analyses including more cases are required to determine more precisely the risk profile for ITP and CVST after a second dose of ChAdOx1 vaccine."*

s

1.1 Assessment of positive and negative findings: I think that the findings relating to CVST, and to a lesser extent ITP, as presented at lines 169-170 and in the Abstract, are overstated. The data are very sparse, and hence IRR estimates for sub-intervals of the 0-27 day risk period lack robustness. Thus, from Table 1, an IRR of 5.01 95% CI (1.16, 21.69) is obtained for the 7-13 day post-vaccination period, yet the IRR for the whole 0-27 day risk period is 1.00 (0.20, 4.92), indicating no increase at all. Unless there are strong a priori reasons to focus on the 7-13 day sub-interval, this contrast suggests lack of robustness, or less compellingly that the vaccine alters the timing of events within the interval. In addition, the number of events in the 7-13 day risk period being ≤ 5 (perhaps only 2 or 3), the asymptotic assumptions upon which the calculation of the confidence interval is based are of questionable validity. This is especially so in view of the (presumably) comparatively large number of parameters being estimated. A more accurate description of the results, in my view, would reflect the tentative nature of the evidence and might go something like this: "No evidence of an increased risk was found for the 0-27 day risk period (IRR 1.00, 95% CI 0-20 to 4.92). However, few (≤ 5) events arose within this risk period. It is perhaps noteworthy that these events all clustered in

the 7-13 day period. Further analyses including more cases are required to determine more precisely the risk profile for CVST after a second dose of ChAdOx1 vaccine".

Response: To better reflect the tentative nature of the evidence, we have amended our abstract to include the recommended text (Line 37):

"No evidence of an increased risk of CVST was found for the 0-27 day risk period (IRR 0.83, 95% CI 0.16 to 4.26). Overall, there were, few (≤5) events within this risk period, and it is perhaps noteworthy that these events all clustered in the 7-13 day period (4.06, 95% CI 0.94 to 17.51)."

And (line 62):

"Further analyses including more cases are required to determine more precisely the risk profile for ITP and CVST after a second dose of ChAdOx1 vaccine."

1.2 Likewise, the authors choose to highlight a "borderline increased risk" of ITP after second dose of ChAdOx1 vaccine, but only in the 14-20 day period. The IRR for the 0-27 day risk period as a whole is 1.16 (0.62, 2.17), which is not suggestive of a real effect; 6 of the 13 events in this risk period arose in the 14-20 day sub-interval. The IRRs suggest that the distribution of the 13 events in the risk period might be 2,3,6,2, which hardly seems like compelling evidence of clustering: unless there is powerful a priori evidence to think that ITP might be clustered 14-20 days after vaccination, this distribution could just as well be a chance effect. At most, the results might suggest that further investigation in more powerful studies should be undertaken.

Response: We agree with the reviewer that an a priori reason is required for the subintervals of interest and have amended the following in our Discussion (Line 229):

"*Although there was a priori evidence that ITP might be clustered between 14 to 20 and 21 to 27 days (with incidence rate ratios of 7.81 and 14.07 respectively[9]) and unusual venous thrombosis events take place 5 to 16 day post ChAdOx1[12], low numbers distributed across the time periods and the use of multiple comparisons mean that this distribution could be a chance effect. Further investigation in studies with greater power is therefore warranted."*

1.3 On the other hand, it is perhaps noteworthy that 6/8 IRRs for the 0-27 day risk period for ChAdOx1 vaccine and 7/8 for BNT162b2 are less than 1. Several of these IRRs suggest a statistically significantly protective effect. For ChAdOx1 vaccine, this is the case for VTE, PE, ATE, and haemorrhagic events; and for BNT262b2, for VTE, DVT, PE, and ATE. This is most likely due to well-vaccinee bias, as people who are unwell defer vaccination. Alternatively, it might conceivably be a by-product of vaccine protection against COVID-19, if COVID is a cause of these events (I presume that COVID infections were not allowed for in the analysis as time-varying confounders). Of all the findings of the paper, these are the most robust. Might they not be worthy of at least brief comment in the Discussion?

Response: We have included the following comments in the Discussion section (Line 185):

"Several IRRs suggest a statistically significantly protective effect for ChAdOx1 and BNT262b2 vaccines. This includes VTE, PE, arterial thromboembolic, and haemorrhagic

events for ChAdOx1; and VTE, DVT, PE, and arterial thromboembolic events for BNT262b2. This is most likely due to well-vaccinee bias, as people who are unwell defer vaccination. Alternatively, this might conceivably be a by-product of vaccine protection against COVID-19, if COVID-19 is a cause of these events [14].”

and (Line 234) *“SARS-CoV-2 infections were included in the analysis as time-varying confounders.”*

2. SCCS assumptions: The methods seem generally sound but would benefit from discussion of the validity of the two key assumptions.

2.1 The first assumption is that occurrence of an event does not lead to the cancellation of vaccination. If such cancellations do in fact occur, then including only cases who have received both vaccine doses, as done here, will oversample events occurring post dose 2, and hence may tend to inflate the IRRs. Since no compellingly positive associations are found, this is not a major issue. (A simpler analysis with observation periods starting at second dose would avoid the issue entirely.)

Response: We now acknowledge this SCCS assumption as a limitation in the Discussion (Line 245):

“We only included cases who received both vaccine doses. This will potentially oversample events occurring post dose two, and hence may have tended to inflate the IRRs”

2.2 The second assumption is that the events of interest do not cause early deaths and thus censor the observation period (deaths from other causes are not at issue). Some comment on this would be appropriate, including mention of the numbers of cases and proportions who died during the observation period.

Response: There were no 0-27 day deaths from second dose ITP and CVST. We have acknowledged this assumption in the Discussion section (Line 246):

“We also assumed that the events of interest did not cause early deaths and thus censored the observation period. There were no deaths 0-27 days post second dose ITP and CVST.

3. Data consistency: The data description and data tabulations sometimes appear inconsistent. For example for CVST and BNT262b2: according to Table 2 there are 9 events in a baseline period. If each of these corresponds to a separate case (as stated on line 333) and all cases receive a second dose (as stated on line 328) then all these 9 cases should have at least one day of exposure to a second dose. Yet the person-time presented in Table 2 suggests that only 6 cases are left at this point.

Response: We have checked the data tabulations and corrected a typo in the Pfizer pulmonary events where we have the AZ figures. We had in fact included the results for all vaccinated individuals, not just those vaccinated with dose 2. We have corrected this and the table now includes only those who got 2 doses of the vaccines. This has no impact on the numbers and person time post dose 2 but the numbers of events and person time in some of the baseline and clearance periods are reduced. This has a slight impact on the IRR’s and we have amended results throughout. This includes an amendment to our Conclusion (Line 258):

"Second dose ChAdOx1 vaccination was associated with borderline increased risk of ITP and CVST events, however these events were rare and usually short-lived"

4. Further points: The following points came to mind while reading the paper.

4.1 Line 140: "this is one of the first real-world contemporaneous studies": another is Jabagi et al (2022), Myocardial infarction, stroke, and pulmonary embolism after BNT162b2 COVID-19 vaccine in people aged 75 years or older. JAMA 2022; 327(1):80-82. Doi:10.1001/jama.2021.21699.

Response: We have now included this study in the Discussion (Line 208):

"One US study found no significant increased risk in those over 75 years 14 days following first or second dose of BNT162b2 vaccine [17]."

4.2 Lines 151-2 Possible shortcomings of the READ codes: is anything known about the positive predictive values of these codes for the events studied? This is relevant because low PPV will bias results towards the null. Also, how accurate are the event timings? This is particularly relevant to the interpretation of timings of events within the 0-27 day risk period.

Response: We do not have PPV for the specific codes used in this analysis – however, as these are major diagnoses they are likely to be recorded accurately, with high completeness and be timely in the EHR. We acknowledge in the Discussion section that information on PPV and accuracy of timing for these codes is not available. If low PPV this will likely to bias results towards the null (Line 221):

"*Major diagnoses are likely to be recorded in a timely way, be accurate and have high completeness [20]. Information on the PPV and accuracy of timing for Read codes was not available. If low PPV occurs, it is likely to have biased results towards the null.*

4.3 Tables 1 and 2: please clarify whether the data given for the baseline and clearance periods relate to all 3 baseline and both clearance periods indicated in Figure 1, or just those adjacent to the dose 2 risk period.

Response: We have clarified these time periods in that the baseline periods contain three baseline periods (prior to dose 1, and before and after dose 2 see Figure 1) in the Tables 1 and 2.

"[a] *Includes all three baseline periods before dose 1 and before and after dose 2 - see Figure 1*
[b] *Second dose clearance period*"

4.4 Tables 1 and 2: The CIs quoted when there are 0 events are (0.00, Inf): this is not strictly correct. When there are 0 events, the CI cannot be calculated using conditional Poisson regression (though other methods could be used), so strictly speaking no CI should be quoted.

Response: We have now removed the CIs in question from Tables 1 and 2 and added the following footnote:

"*NA – Not Applicable as when there are 0 events, the CI cannot be calculated using conditional Poisson regression"*

<u>Reviewer #2:</u>

This is a brief report on the risk of thromboembolic and thrombocytopenic adverse events following second doses of AZ and BNT in a nationwide Scottish population. The authors utilize a SCCS approach and observe an association between AZ and CVST in the 7 to 13 days period following the second dose. I would like the authors to elaborate on the following issues.

1. The VITT signal was identified in younger females. This study does not really evaluate the risk of a second dose of AZ in younger females. Please discuss the relevance of these results to the VITT signal in more detail. The authors found 0 cases of CVST in <30 yr olds. Is it possible to evaluate how strong a signal could have been detected given the number of <30 yr old females in Scotland with a second dose of AZ?

Response: We have included information on the number of females <30 years old with a second dose of AZ and have amended our conclusions with regard to age and CVST presentation (and agree that further better powered studies are required - see response to Reviewer 1, Point 1.2) (Line 90):

"*Between 8 December 2020 and 7 November 2021, 2.0 million people (39,682 females and 33,588 males under 30 years of age) were vaccinated with a second dose of ChAdOx1 and 1.6 million people (294,511 females and 276,476 males under 30 years of age) with a second dose of BNT162b2 (Extended Data Figure 1)."*

We have removed age from our Abstract and Conclusions (Line 46 & Line 259):

"*Second dose ChAdOx1 vaccination was associated with increased borderline risks of ITP and CVST events. with an increased risk of CVST events ~~in older females~~ and borderline increased risk of ITP*"

And acknowledge that studies with greater power are required (Line 233):

"Further investigation in studies with greater power is therefore warranted"

2. Please address multiple comparisons. The authors have conducted a large number of comparisons including using multiple risk periods. Would it not have made more sense to pre-specify a main risk period based on the VITT signal, alternatively a 28-day period? In the 28-day period following AZ2 the CVST association was 1.00 (0.20-4.92). The 7-13 day association could be a random finding.

Response: Please see our response to Reviewer 1 (see point 1.2 repeated below for your convenience) where we have acknowledged in the Discussion section our a priori reasoning for selecting these time periods and also acknowledge that low numbers distributed across the time periods mean that any distribution found could be a chance effect and that further investigation in more powerful studies is therefore warranted. We also acknowledge that the use of multiple comparisons of time periods may have led to a chance effect (Line 229):

"*Although there was a priori evidence that ITP might be clustered between 14 to 20 and 21 to 27 days (with incidence rate ratios of 7.81 and 14.07 respectively[9]) and unusual venous thrombosis events take place 5 to 16 day post ChAdOx1[12], low numbers distributed across the time periods and the use of multiple comparisons mean that this distribution could be a chance effect. Further investigation in studies with greater power is therefore warranted."*

3. Discuss why a CVST association is observed in the clearance period. This is puzzling to me. Who would receive a CVST diagnosis and go on to be vaccinated in the next 2 weeks.

Response: This scenario is a possibility and therefore we have included a new section regarding vaccination post thrombosis events in the Discussion section (Line 248):

"The scenario of CVST being observed in the clearance period prior to second dose ChAdOx1 is possible. As CVST during the clearance period will not be due to the first dose ChAdOx1 vaccination (due to the gap in vaccination doses) and vaccination is recommended for individuals, even with past clotting episodes, as they remain at risk of COVID-19 disease[20]."

4. What is the distribution of the dose-interval between AZ1 and AZ2 in the Scottish setting?

Response: We have included Figure 3 in the Appendix to show the dose-interval distribution between AZ1 and 2.

5. Please argue (using sensitivity analyses) that the assumptions of the SCCS approach is fulfilled. E.g. is it likely that an outcome event does not influence future vaccination propensity?

Response: This is the same point as reviewer 1 point 2.1. To account for the occurrence that an event might have reduced the likelihood of vaccination we included a pre-second dose risk clearance period (Line 725).

"*A 14-day clearance period was used prior to the exposure date (Figure 1)."*

We also acknowledge this assumption as a limitation of our analysis in the discussion (Line 245):

"We only included cases who received both vaccine doses. This will potentially oversample events occurring post dose two, and hence may have tended to inflate the IRRs"

6. The authors use READ codes from the GP setting (as I understand the manuscript) with follow up in hospital data. Would it not make more sense to use the hospital data to identify something like CVST or pulmonary embolism? Presumably cases are missed by using GP data? 7. Have these READ codes been validated for thromboembolic- and thrombocytopenic outcomes?

Response: We have acknowledged in the discussion that the accuracy/PPV of specific event codes is not available - see response to Reviewer #1, 4.2

The reason for using the GP data is that there is a delay in hospital codes and there we have included the following as a study limitation in the Discussion section (Line 228):

"*Due to delays in reporting of coded data, we did not use hospital coded data for our primary analysis* [21]."

We have added the following to the Methods section in regard to the Read code lists (Line 704):

"*The code lists used in this study were drawn up by the EAVE II clinical team and validated by experts in neurology and haematology.*"

8. How was incidences calculated? This is not described in the Methods.

Response: We have now explained how this was calculated in the Methods section (Line 706): When we discuss incidence in the results we mean the number of events in the whole exposure period 28 days post dose 2 and expressed in terms of the number vaccinated.

"*Incidence was the outcome of interest during the post vaccine exposure periods after vaccination; the primary incidence covered the 28 day period post second dose and is expressed as number of events per 100,000 vaccinated.*"

We also clarify that in the SCCS were are modelling incidence rate which is the number of events divided by the person time exposure in the exposure groups (Line 710).

"Incidence rates of the outcomes in the different exposure periods were calculated as the number of events in the exposure period divided by the person time in the exposure period."

9. Discuss these incidences in contrast to what has been observed after AZ1.

Response: We have now added the following to the Discussion section (Line 208):

"*One US study found no significant increased risk in those over 75 years 14 days following first or second dose of BNT162b2 vaccine* [17]*. The incidence of ITP post 0-28 day second dose ChAdOx1 was lower than post first dose (0.59 vs. 1.13 per 100,000 doses)* [9]*. Post-second dose ChAdOx1 incident CVST events were similar (0.05 per 100,000 doses vs. 0.44 to 0.59 incident CVST cases per million people)*[16]

10. L340-342: I did not understand how this was done or what it was used for.

Response: We have added the following clarification (Line 700):

"*Read codes (Version 2) were used to determine adverse incident events recorded in the primary care EHR data (Supplementary Table 1), which were then followed up for whether hospitalizations or deaths occurred in the linked Rapid Preliminary Inpatient Data (RAPID) dataset and National Records Scotland for hospitalization and mortality outcomes, respectively.*

<u>**Reviewer #3:**</u>

The study reports on the incidence of adverse events after the second dose of COVID-19 vaccines. The data are of interest but the limited information on the details of the few patients who developed these adverse events make it very difficult to draw further conclusions.

Response: Thank you for these comments and we agree that the study is of interest.

1. Page 2, line 32-32: Did the individuals receive both ChAdOx1 as first and BNT162b2 as second dose, as implied by "and"? Probably "or" would be more clear.

Response: We have amended this line in the Abstract and replaced 'and' with 'or'.

2. Page 3, line 56: Refs 6, 7, 8 do not report on ITP but on VITT

Response: We have corrected this text and accompanying references.

3. Page 4. Line 79: please explain IRR

Response: We have now spelt out incidence rate ratio (IRR)

4. Abstract, line 34-35 states that ChAdOx1 is associated with a borderline increased risk for ITP, Page 4, line 78 states that there is no increased risk for thrombocytopenia. This is confusing.

Response: We have more consistently expressed findings/interpretation in these 2 sections and indeed throughout the paper. The borderline increased risk of ITP finding is located on Page 4 Line 85.

5. Page 5, line 93-94: 1310 and 10077% are likely an error

Response: Thank you for pointing out this error, which has now been corrected.

6. Page 5, line 95-96: There are only two drugs, which induce ITP, i.e. gold and alemtuzumab. It would be very unusual if the patients did receive these two drugs. Please do not confuse ITP with drug dependent thrombocytopenia, which is a very different mechanism although both are antibody mediated.

Response: We have included this clarification (Line 123):

"*Sixty-five percent of post-second dose ChAdOx1 ITP cases had prior prescriptions that could induce drug dependent thrombocytopenia or ITP, compared with 51% of those who were unvaccinated at the time of their ITP event."*

7. Page 5, last para: this observation is highly interesting. CVST without thrombosis argues against VITT/TTS as underlying cause and hints towards the possibility that the vaccine itself is altering the cerebral veins. Any information on the platelet counts in these patients would be important, either known baseline platelet counts or a potential increase in platelet counts after treatment. Are there any data on the incidence of VITT/TTS? At the time of the second dose anti-PF4 antibodies have widely been tested in patients with adverse events after vaccination.

Response: We agree this would be of interest, however, it is not currently possible to determine whether VITT/TTS was the underlying cause of CVST as we do not have access to anti-PF4 antibody data nor are we able to access platelet counts from the hospital data (all CVST were hospitalised for at least a week at the time of the event). We have now expanded the Discussion section to clarify this (Line 235):

"*It was also not possible to determine whether VITT/TTS was the underlying cause of CVST as we do not have access to anti-PF4 antibody data nor are we able to access platelet counts from the hospital data (all CVST were hospitalised for at least a week at the time of the event).*"

8. Page 7, line 149-150: it is striking that adverse events after vaccination with ChAdOx1 in LIMCs are very rarely reported. It is still unclear whether this is related to underreporting or to different susceptibility. Therefore wording related to generalizability should be more careful

Response: We agree and have added a proviso to this statement (Line 216):

"We believe our findings are likely to have generalizability across countries using these vaccines as part of national vaccination programs (with the proviso that further work is needed to determine if low adverse events rates in low- and middle-income countries is a result of underreporting or different susceptibility)."

Editorial feedback

Please complete or update the following checklist(s) to verify compliance with our research ethics and data reporting standards. Address all points on the checklist, revising your manuscript in response to the points if needed.
The form(s) must be downloaded and completed in Adobe Reader rather than opened in a web browser. Each form must be uploaded as a Related Manuscript file at the time of resubmission.

Editorial policy checklist:
https://www.nature.com/documents/nr-editorial-policy-checklist.pdf

Reporting summary:
https://www.nature.com/documents/nr-reporting-summary.pdf

DATA AND CODE AVAILABILITY
*** All Nature Communications manuscripts must include a "Data Availability" section after the Methods section but before the References. If any of the data can only be shared on request or are subject to restrictions, please specify the reasons and explain how, when, and by whom the data can be accessed. For more information on this policy and a list of examples, see:**
https://www.nature.com/documents/nr-data-availability-statements-data-citations.pdf

*** Please also include a "Code Availability" section after the "Data Availability" section. If the code can only be shared on request, please specify the reasons. For more information on our code sharing policy and requirements, please see:**
https://www.nature.com/nature-portfolio/editorial-policies/reporting-

standards#availability-of-computer-code

*** We strongly encourage you to deposit all new data associated with the paper in a persistent repository where they can be freely and enduringly accessed. We recommend submitting the data to discipline-specific and community-recognized repositories; a list of repositories is provided here: http://www.nature.com/sdata/policies/repositories Refer to our data policies here: https://www.nature.com/nature-portfolio/editorial-policies/reporting-standards#availability-of-data**

*** To maximise the reproducibility of research data, we strongly encourage you to provide a file containing the raw data underlying the following types of display items:**
- Any reported means/averages in box plots, bar charts, and tables
- Dot plots/scatter plots, especially when there are overlapping points
- Line graphs
The data should be provided in a single Excel file with data for each figure/table in a separate sheet, or in multiple labelled files within a zipped folder. Name this file or folder 'Source Data', and include a brief description in your cover letter. The "Data Availability" section should also include the statement "Source data are provided with this paper."

Response: We have completed the required checklists and included information on data and code availability (see line 691).

The opportunity to respond to the valuable feedback from reviewers has helped us to improve the quality of our work, for which we are most grateful. We trust that these revisions are to your satisfaction. Please do not however hesitate to contact us if you require any further clarifications or revisions.

With kind regards,

Aziz Sheikh, Colin Simpson and Chris Robertson, on behalf of the co-authors

Additional references

17 Jabagi, M. J. *et al.* Myocardial Infarction, Stroke, and Pulmonary Embolism After BNT162b2 mRNA COVID-19 Vaccine in People Aged 75 Years or Older. *JAMA* 327, 80-82, doi:10.1001/jama.2021.21699 (2022).

21 Public Health Scotland. *SMR Timeliness*, <https://www.isdscotland.org/products-and-Services/Data-Support-and-Monitoring/SMR-Timeliness/>

22 UK Government. *COVID-19: the green book, chapter 14a*, <https://www.gov.uk/government/publications/covid-19-the-green-book-chapter-14a> (2022).

22 UK Government. *COVID-19: the green book, chapter 14a*, <https://www.gov.uk/government/publications/covid-19-the-green-book-chapter-14a> (2022).

Reviewers' Comments:

Reviewer #1:
Remarks to the Author:
The authors have responded to all the comments I made in my original review, and have amended the paper and its abstract accordingly.

I have no further major comments. Just one very minor one: at Line 149, "One US study" should be amended to "One French study".

In my view the paper is suitable for publication.

Paddy Farrington

Reviewer #2:
Remarks to the Author:
Thank you for the careful revision. I have nothing further to add.
